# Supplementary figures and images for: PACSIN2 as a modulator of autophagy and mercaptopurine cytotoxicity: mechanisms in lymphoid and intestinal cells
Source: Life Sci Alliance. 2023 Jan 3;6(3):e202201610. doi: 10.26508/lsa.202201610 (PMC9811133; doi:10.26508/lsa.202201610)

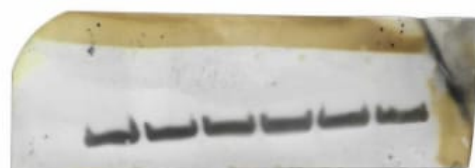

Supplement: Supplementary file 1 [file LSA-2022-01610_SdataF3.1.pdf]

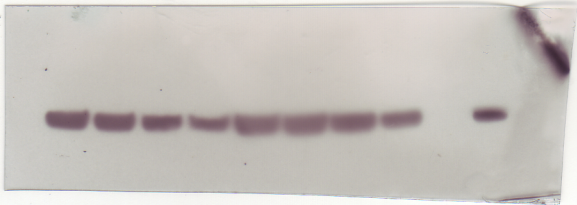

Supplement: Supplementary file 2 [file LSA-2022-01610_SdataF3.2.pdf]

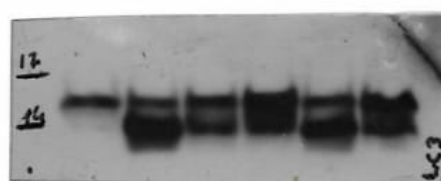

Supplement: Supplementary file 3 [file LSA-2022-01610_SdataF3.3.pdf]

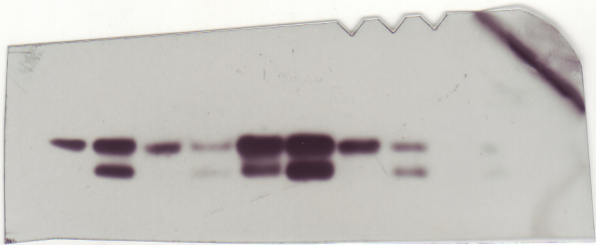

Supplement: Supplementary file 4 [file LSA-2022-01610_SdataF3.4.pdf]

20100309

# IP PACSIN2

# IP LC3

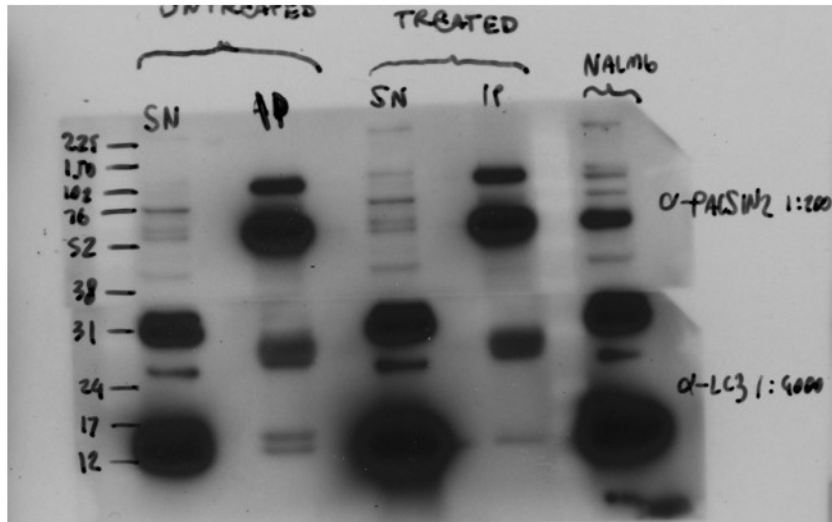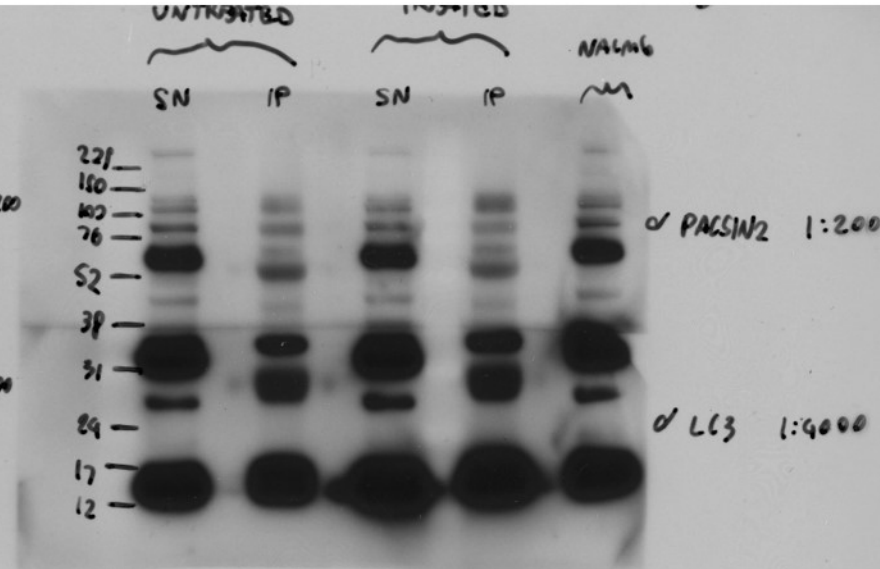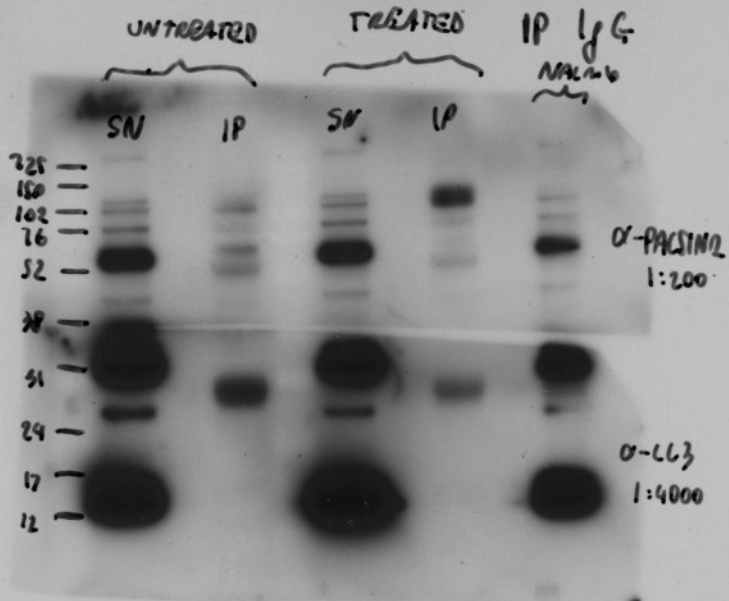

Supplement: Supplementary file 5 [file LSA-2022-01610_SdataF6.pdf]

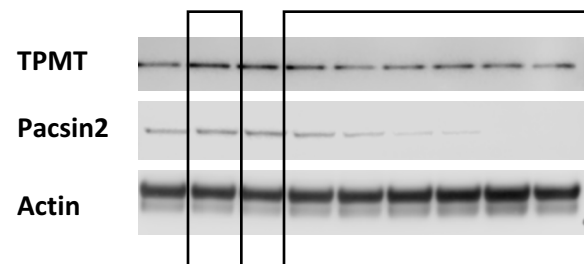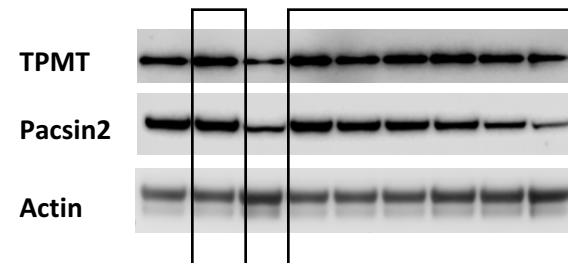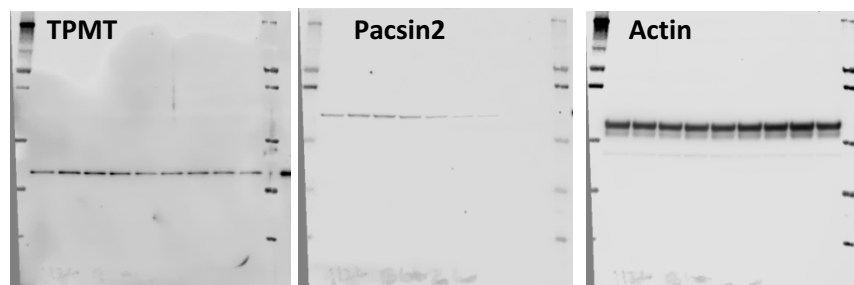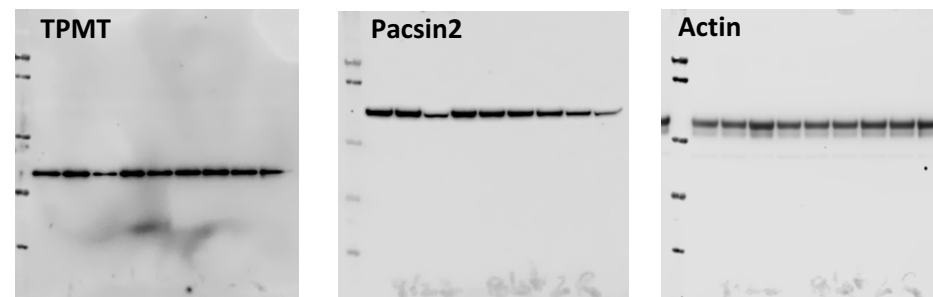

Supplement: Supplementary file 6 [file LSA-2022-01610_SdataF10.pdf]
