# Supplementary material for: PACSIN2 as a modulator of autophagy and mercaptopurine cytotoxicity: mechanisms in lymphoid and intestinal cells
Source: Life Sci Alliance. 2023 Jan 3;6(3):e202201610. doi: 10.26508/lsa.202201610 (PMC9811133; doi:10.26508/lsa.202201610)
Supplement: Supplementary file 7 [file LSA-2022-01610_TableS1.docx]

| **Cell line** | **Treatment concentration range** | **Treatment duration interval** | **Aim** | **Assay** | **Results described in figure** |
| --- | --- | --- | --- | --- | --- |
| MEFs cell line | rapamycin (50 µg/ml); cycloheximide (5 µg/ml) | 0, 4, 8, 12 and 24 hours | to evaluate PACSIN2 protein stability after autophagy induction | immunoblotting | Figure 2 |
| NALM6 cell line and LS180 cell line | chloroquine (30 µM) | 4 hours or 24 hours | to investigate the autophagic flux | immunoblotting | Figure 3 |
| NALM6 cell line | chloroquine (50 µM) | 3 hours | to investigate the autophagic flux | co-immunoprecipitation | Figure 6 |
| NALM6 cell line | mercaptopurine (from 0.313 µM to 20 µM) | 72 hours | to evaluate drug sensitivity | MTT drug sensitivity assay | Figure 7 |
| NALM6 cell line | tunicamycin (from 0.0005 to 50 μM) | 72 hours | to evaluate unfolded protein stress response | MTT drug sensitivity assay | Figure 7 |
| LS180 cell line | mercaptopurine (from 0.313 µM to 160 µM) | 72 hours | to evaluate drug sensitivity | MTT drug sensitivity assay | Figure 7 |
| LS180 cell line | tunicamycin (from 0.05 to 156 μM) | 72 hours | to evaluate unfolded protein stress response | MTT drug sensitivity assay | Figure 7 |
| LS180 cell line | mercaptopurine (1.25 µM and 2.5 µM) | 24 hours, 48 hours | to evaluate autophagy induction | immunoblotting | Figure 8 |
| LS180 cell line | mercaptopurine (1.25 µM and 2.5 µM); doxorubicin (5 µM, positive control) | 24 hours, 48 hours | to evaluate apoptosis induction | immunoblotting | Figure 9 |
| LS180 cell line | mercaptopurine (1.25 µM and 2.5 µM); doxorubicin (5 µM, positive control) | 24 hours, 48 hours and 72 hours | to evaluate mitochondrial membrane potential alterations | DiOC6 assay | Figure 9 |
| LS180 cell line | mercaptopurine ( 2.5 µM) | 24 hours, 48 hours | to evaluate thiopurines metabolites | HPLC-UV analysis | Supplementary figure 7 |
| NALM6 cell line | cycloheximide (100 µg/ml) | from 15 minutes to 72 hours | to evaluate the impact of *PACSIN2* KD on TPMT protein stability | immunoblotting | Figure 10 |

**Table 1 supplementary**. Summary of cell line treatments
